# The attention schema theory: a mechanistic account of subjective awareness

**DOI:** 10.3389/fpsyg.2015.00500

**Published:** 2015-04-23

**Authors:** Michael S. A. Graziano, Taylor W. Webb

**Affiliations:** Princeton Neuroscience Institute, Psychology Department, Princeton UniversityPrinceton, NJ, USA

**Keywords:** consciousness, awareness, attention, control theory, attention schema, body schema, biased competition

## Abstract

We recently proposed the attention schema theory, a novel way to explain the brain basis of subjective awareness in a mechanistic and scientifically testable manner. The theory begins with attention, the process by which signals compete for the brain’s limited computing resources. This internal signal competition is partly under a bottom–up influence and partly under top–down control. We propose that the top–down control of attention is improved when the brain has access to a simplified model of attention itself. The brain therefore constructs a schematic model of the process of attention, the ‘attention schema,’ in much the same way that it constructs a schematic model of the body, the ‘body schema.’ The content of this internal model leads a brain to conclude that it has a subjective experience. One advantage of this theory is that it explains how awareness and attention can sometimes become dissociated; the brain’s internal models are never perfect, and sometimes a model becomes dissociated from the object being modeled. A second advantage of this theory is that it explains how we can be aware of both internal and external events. The brain can apply attention to many types of information including external sensory information and internal information about emotions and cognitive states. If awareness is a model of attention, then this model should pertain to the same domains of information to which attention pertains. A third advantage of this theory is that it provides testable predictions. If awareness is the internal model of attention, used to help control attention, then without awareness, attention should still be possible but should suffer deficits in control. In this article, we review the existing literature on the relationship between attention and awareness, and suggest that at least some of the predictions of the theory are borne out by the evidence.

## Introduction

What is subjective experience, and how could it possibly result from the activity of the brain? Does it serve any useful purpose or is it merely an epiphenomenon arising from the activity of the brain without playing a role in its function? We recently proposed the attention schema theory in an attempt to answer these and other related questions (Graziano and Kastner, 2011; Graziano, 2013; Graziano and Webb, 2014; Kelly et al., 2014). In a nutshell, the theory proposes that subjective awareness is the brain’s internal model of the process of attention.

**FIGURE 1:**
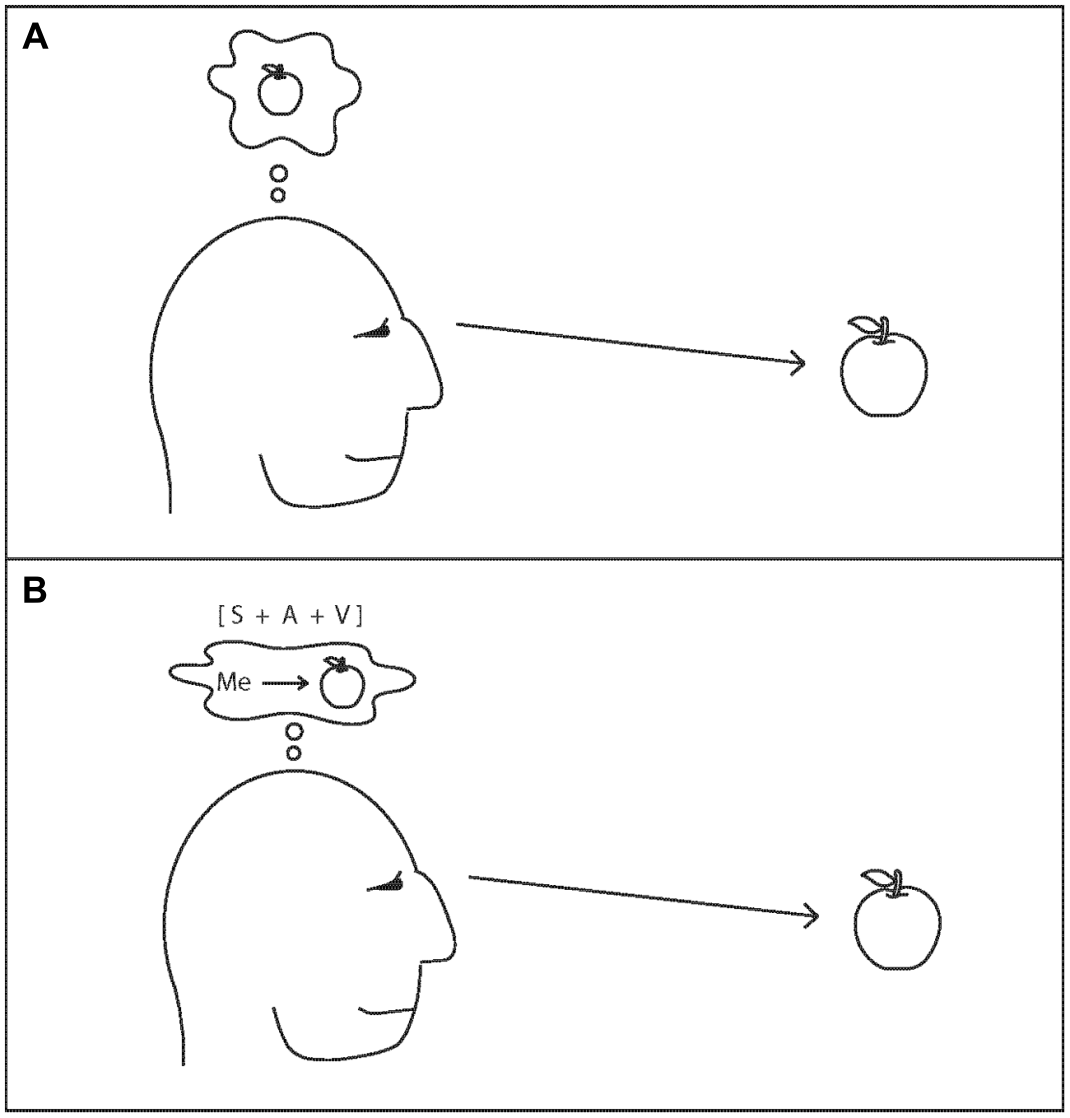
**The attention schema theory. (A)** Visual attention is captured by the image of an apple. On its own, this process results in the ability to accurately process the stimulus features – shape, color, motion, etc. – of the apple, but it does not provide any basis for the brain to conclude that it possesses subjective awareness of the apple. **(B)** In order for the brain to conclude that it possesses subjective awareness of the apple, the brain requires more than just information about the visual stimulus [V]. It requires that the brain also have information about the self [S], and about the process that links the two together, attention [A], such that the larger, overarching relationship between self, attention, and stimulus [S+A+V] can be represented. According to the theory, the A component of this larger representation would not include any of the physical, mechanistic details of the real process of attention, and so it would appear to depict a physically impossible entity, a process that can accomplish the same things as attention without the mechanistic basis for doing so. This brain would conclude that it possesses a fundamentally mysterious property: a mental possession of something, a subjective awareness. In this account, the brain’s conclusion that it has subjective awareness reflects the information contained in a simplified but useful model of attention, an attention schema.

**Figure 1** illustrates the main components of the attention schema theory. The person in **Figure 1A** is attending to an apple. The visual representation of the apple has won a competition, suppressing the representations of other visual stimuli. This account of the brain basis of attention is commonly referred to as the biased competition theory of attention (Desimone and Duncan, 1995; Beck and Kastner, 2009). The outcome of this signal competition has important consequences. Attended stimuli, such as the apple in **Figure 1A**, exert a much greater influence than unattended stimuli on other brain systems and therefore on memory and on behavior.

The apple, however, is only one part of a larger whole. **Figure 1B** illustrates a brain that has constructed a model of this larger whole. The model contains not only the visual representation of the apple (V), but also a model of the self as a physical and mental agent (S), and a model of the relationship between them: attention (A).

How does this proposal address the brain basis of subjective experience? The hypothesized model of attention, or the attention schema (component A in **Figure 1B**), would not be a perfectly detailed model of the neuroscientific phenomenon of attention. It would not include anything about lateral inhibition, signal competition, or action potentials. The brain has no functional use for information about those physical details. Instead, the model would be more like a cartoon sketch that depicts the most important, and useful, aspects of attention, without representing any of the mechanistic details that make attention actually happen.

Based on the information contained in this simplified model, brain B would conclude that it possesses a phenomenon with all of the most salient aspects of attention – the ability to take mental possession of an object, focus one’s resources on it, and, ultimately, act on it – but without any of the mechanisms that make this process physically possible. It would conclude that it possesses a magical, non-physical essence, but one which can nevertheless act and exert causal control over behavior, a mysterious conclusion indeed.

According to this theory, there is, of course, no actual mystery. Attention does have a real physical basis, but the mechanistic details of the process of attention are not included in the only relevant information to which the brain has access. The attention schema theory can therefore explain why a brain would *conclude* that such a mystery exists. The internal model of the self (S) includes information about the body and can therefore lead to reports about the physical structure of the body, but not reports about awareness. The visual representation of the apple (V) contains information about that apple and can therefore lead to reports about that apple, but not reports about awareness. The hypothesized attention schema (A), however, contains information about the way the brain attends, processes information, and facilitates action. The information in that attention schema leads the brain, in this hypothesis, to *conclude* it has awareness.

This view, that the problem of subjective experience consists only in explaining why and how the brain *concludes* that it contains an apparently non-physical property, has been proposed before (Dennett, 1991). The attention schema theory goes beyond this idea in providing a specific functional use for the brain to compute that type of information. The heart of the attention schema theory is that there is an adaptive value for a brain to build the construct of awareness: it serves as a model of attention.

As a comparison, consider the construct of color. Objects in the world around us reflect light in a complex spectrum. The brain, however, deals in the simpler, computed property of color. The property of color is computed in specialized networks in the visual system, and that information is linked to or integrated with information about the shape and location of objects. In that way, accessing those visual models, a brain has sufficient information to report that this apple is red or that car is blue.

Just so, in the present theory, the physical reality is attention, whereas the brain computes the simpler construct of awareness. Brain B in **Figure 1** has constructed a model that depicts itself, a model that depicts the apple, and a model that depicts awareness, and it has linked those models together. As a result, it has sufficient information to report that it is aware of the apple.

In another sense, however, the construct of awareness is different from the construct of color. Color is a sensory construct. A color has a specific, precise location, often a sharp border, and can have brightness and saturation, which are precisely definable quantities. Awareness, as a model of attention, would have none of those tangible properties. Attention has no highly precise location. It is vaguely inside the head. It is invisible. It has no brightness or saturation. The item being attended may have brightness, but not the act of attention itself. Awareness, as a model of attention, would present a picture of something vague, without sharp borders, without tangible attributes, and yet still something real or potent that can be attached to other items.

In the attention schema theory, attaching the construct of awareness to a specific item – whether an apple, or a thought, or anything else – requires some method of integrating information across disparate brain areas into a single, larger, brain-spanning representation (the S+A+V of **Figure 1B**). In this sense, the attention schema theory resembles many previous proposals in which consciousness depends on an integration of information, a binding of information, a brain-wide global workspace, or a settling of networks into a single coherent state (Baars, 1983; Crick and Koch, 1990; Tononi, 2008; Schurger et al., 2012). The attention schema theory is consistent with these previous proposals, but also goes beyond them. In the attention schema theory, awareness does not arise just because the brain integrates information or settles into a network state, any more than the perceptual model of color arises just because information in the visual system becomes integrated or settles into a state. Specific information about color must be constructed by the visual system and integrated with other visual information. Just so, in the case of awareness, the construct of awareness must be computed. Then it can be integrated with other information. Then the brain has sufficient information to conclude and report not only, “thing X is red,” or, “thing X is round,” but also, “I am *aware* of thing X.”

The purpose of the present review is to provide some background for the attention schema theory, focusing on the relationship between attention and awareness. We begin by defining in what sense we use the terms attention and awareness. We also briefly discuss the logic of model-based control – the extent to which a model is useful for controlling the thing that is being modeled, in this case the usefulness of an attention schema in controlling attention. We illustrate this point with reference to the role of the body schema in controlling the body.

The attention schema theory makes a testable prediction. If awareness is an internal model of attention and is used to help control attention, then without awareness, attention should still be possible but it should suffer deficits in control. To assess whether the attention schema theory is supported by the existing evidence, we review the experimental literature concerning the relationship between attention and awareness. Although attention and awareness are typically highly correlated, a large body of evidence now shows that they can be dissociated. Moreover, at least some studies suggest that without awareness, attention suffers from some loss of normal control. We argue that this relationship supports the attention schema theory.

Finally, we argue that the attention schema theory accounts in a natural way for one of the most puzzling and mysterious aspects of awareness, the fact that we can become aware of both external and internal events. Because attention can be directed to both external and internal information, one would expect a model of attention to encompass both of these possibilities as well.

Although the present review focuses on the role that awareness may play in the control of attention, the attention schema theory has a broader scope. In the theory, awareness, as an internal model of attention, may play a critical role in social cognition (modeling the attentional states of others), in religiosity (attributing awareness to non-physical beings), in integrating disparate types of information (such as, in **Figure 1B**, integrating a self model with external visual information), and so on. These other aspects of the attention schema theory have been discussed in detail in other places (Graziano and Kastner, 2011; Graziano, 2013; Graziano and Webb, 2014; Kelly et al., 2014).

## Defining Attention and Awareness

A common claim is that “everyone knows” what attention and awareness are. On closer examination, however, the usage of these terms turns out to be quite inconsistent. Here, we define the senses in which the terms have commonly been used and in which we use them. Our goal here is to clarify our own meaning, not to try to impose any normative use of those words.

## Attention

Most definitions of ‘attention’ have something to do with the selective processing of certain pieces of information more than others. Because the amount of information with which our senses are bombarded is typically far too vast to deeply process in its entirety, some mechanism must exist to determine or ‘select’ which information to process deeply. Much work in cognitive psychology and neuroscience over the past half century has focused on which factors determine this ‘selection’ process and how the brain accomplishes such an operation.

An influential theory put forward by Desimone and Duncan (1995), the ‘biased competition’ theory, characterizes attention as a signal competition within the brain. Signals compete in order to be more deeply processed and ultimately to influence and guide behavior. This signal competition emerges at the earliest stages of processing in the nervous system and is present at every stage. Competitive processing mechanisms exist, for instance, even within the circuitry in the eye (Kuﬄer, 1953; Hartline et al., 1956) and are present in the primate visual cortex (Moran and Desimone, 1985; Reynolds et al., 1999; Kastner and Ungerleider, 2000). Different factors can influence or ‘bias’ the outcome of this competition. One such factor has to do with the saliency of the stimulus. Especially intense or salient stimuli can ‘grab’ attention in a bottom–up, stimulus-driven manner.

As signals progress through the nervous system, they are increasingly subject to the influence of top–down, biasing signals. By this method, attention can be internally directed, slanting the outcome of this signal competition in a goal-directed manner based on the demands of the current task. Signals that correspond to current goals can be boosted and irrelevant signals can be suppressed. The term ‘attention’ has frequently been used to refer only to these top–down control mechanisms, but we use the term to refer to the entire phenomenon outlined here, ranging from the simple, competitive mechanisms driven by stimulus salience to sophisticated, top–down control mechanisms. To attend to a stimulus is to have its representation win a competition, thus gaining greater signal strength, thus being more likely to influence other brain systems such as those involved in decision-making, movement control, and memory.

There are some important differences between the properties of top–down and bottom–up attentional effects (Posner, 1980; Jonides, 1981). Bottom–up attentional effects, those that are driven by salient stimuli, are task-irrelevant, and their effect on attention is very briefly facilitatory followed by a period during which the effect is briefly inhibitory. Top–down attentional effects, those that are sensitive to task demands or current goals, are by definition task-relevant, and they can have a much more sustained facilitatory effect on attention. Some authors have recently argued that the traditional top–down versus bottom–up distinction in attention research is a flawed dichotomy because some effects do not fall neatly into either category (Awh et al., 2012; Zhao et al., 2013). We are sympathetic to these views. For the purposes of this review, however, we are primarily concerned with the distinction between task-relevant and task-irrelevant effects on attention. The brain must control attention in a task-relevant fashion, and that control of attention, according to the principles of control engineering, can be improved if the brain constructs a model of attention. That model, as described below, should contain information about the dynamics and consequences of attention.

## Awareness

The term ‘awareness’ is arguably even more problematic and subject to multiple definitions than the term ‘attention.’ Many authors, for instance, draw a distinction between different varieties of awareness (Block, 1995; Lau, 2008). Here, we use the terms ‘awareness,’ ‘consciousness,’ and ‘subjective experience’ to mean the same thing. We have in mind a functional, materialist definition of the term ‘awareness.’ It is probably best illustrated in terms of how awareness is measured.

There are two common ways of measuring awareness, and therefore two experimental concepts of awareness: objective and subjective awareness. In objective awareness, a participant is tasked with making an objective discrimination about a stimulus – what color it is, what shape it is, or what side of space it is on, for instance – and to the extent that the participant can make this discrimination at above-chance levels, they are said to be objectively aware of the stimulus. Whether or not the participant feels subjectively that they have perceived the stimulus, or whether they regard their responses as ‘guesses,’ is irrelevant to the psychologist’s notion of objective awareness. This is exactly the opposite with subjective awareness. Subjective awareness is defined precisely as whether or not the participant, in his or her own opinion, has perceived the stimulus. A common way of assessing this is to give an objective discrimination as described above, and then to ask participants if they ‘saw’ the stimulus or were just guessing.

The distinction between objective and subjective awareness is an important one because there are dissociations between the two measures. In the neurological condition known as blindsight, for instance, patients can profess a complete lack of subjective awareness for stimuli in the affected region of space, but at the same time show objective awareness in the sense of making above-chance discriminations about those stimuli, sometimes even at very high levels of accuracy (Weiskrantz, 1986, 1999). The patients regard these discriminations as ‘guesses,’ demonstrating a strong dissociation between objective and subjective awareness. Similar dissociations exist in the condition known as hemispatial neglect, in which patients lose subjective awareness for an entire half of space but retain objective awareness in the sense of showing evidence that they processed information about stimuli in the neglected half of space (Marshall and Halligan, 1988). Additionally, even in normal participants, subjective and objective awareness can be manipulated separately (Lau and Passingham, 2006).

The attention schema theory aims to explain the nature and possible function of subjective awareness, the component that is lost in blindsight and neglect. Throughout this review, we use the terms ‘awareness,’ ‘consciousness,’ and ‘subjective experience’ interchangeably to refer to the concept of subjective awareness, unless otherwise noted.

Although many experimental approaches to consciousness ask whether awareness is present or not, this yes/no dichotomy is obviously a simplification. To address the issue, in some paradigms, subjective awareness is assessed using continuous measures, such as confidence ratings, the perceptual awareness scale (PAS), or post-decision wagering (Overgaard et al., 2006; Fahle et al., 2011; Naber et al., 2011; Szczepanowski et al., 2013). A continuous scale for awareness is compatible with the attention schema theory. Attention can be graded, with more or less attention focused on an item, and therefore awareness, the internal model of attention, should also be graded.

## Model-Based Control

In order to control a complicated system, it is useful to construct a simple model of the system. The idea is a key insight of control theory (Camacho and Bordons Alba, 2004), a branch of engineering concerned with the control of complex systems. Here, we briefly illustrate the logic of this idea, often referred to as model-based control, as it relates to brain mechanisms for controlling the body.

Consider the task of successfully moving one’s arm to grasp an object. One strategy for accomplishing this task is simply to try out a set of muscle forces and reinforce those that result in success. An alternative strategy is to compute a simplified model of the relationship between muscle forces in the arm and the resulting movements. This latter strategy, model-based control, has been shown to play an important role in motor control (Shadmehr and Mussa-Ivaldi, 1994; Mazzoni and Krakauer, 2006; Schaefer et al., 2011). Model-based control is especially dependent on having a relatively accurate model of the body’s current configuration. This model, referred to as the body schema, has a few important properties.

First, the body schema appears to be a simplified, and therefore sometimes inaccurate, model of the body’s configuration. Converging evidence from both psychology and neurophysiology suggests that the brain relies on a set of relatively robust, but ultimately limited, tricks in order to compute the configuration of the body (Graziano and Botvinick, 2002). Though these tricks tend to work well under normal circumstances, laboratory scenarios can be devised that result in the dissociation of the body schema and the actual configuration of the body.

**FIGURE 2:**
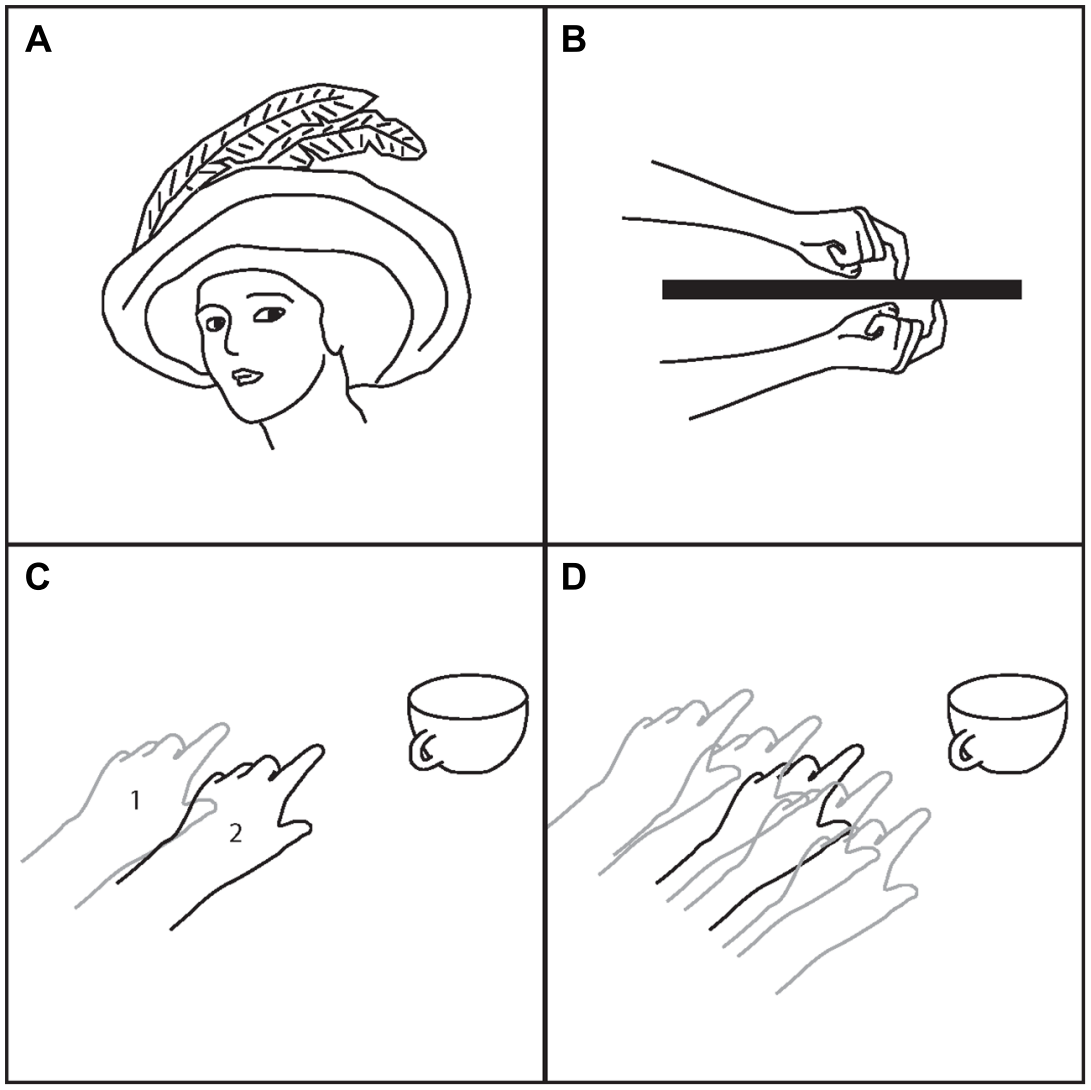
**Examples of model-based control. (A)** To enter a car without hitting your head requires the body schema to include an accurate model of the shape of your head. Women who wore feathered hats would develop an altered body schema to incorporate the hat, and would avoid hitting the feathers (Head and Holmes, 1911). **(B)** The body schema depends on visual and somatosensory input, among other sources of information. Somatosensory input, however, is less accurate than visual. If you put your left hand under the table and leave it for a few minutes, the body schema begins to lose a precise representation of arm position. It is then difficult to point with the right hand, on top of the table, to the exact location of the left hand. **(C)** If the hand is perturbed from point 1 to point 2, but the body schema does not register that change, reaching becomes inaccurate. In this case, the real position is closer to the cup than the position registered by the body schema. The result will be an overreach that may knock over the cup. **(D)** In some cases the body schema loses specificity. The position of the arm is not precisely represented. In that case maintaining a steady arm position is difficult, especially in the face of external, perturbing forces, and reaching accurately is no longer possible.

Second, this dissociation between the brain’s model of the body and the actual body has important consequences for the control of the body (Scheidt et al., 2005; examples of model-based control of the body shown in **Figure 2**). Suppose this internal model is misinformed about the location of the arm. In attempting to reach to a particular location, the motor system will select a set of muscular forces consistent with the arm’s incorrectly represented starting position. The result will be an error in moving the arm to the new position. Or suppose the brain’s internal model of the arm has lost specificity; for example, the sensory nerves of the arm are numbed and the arm is blocked from sight. In that case, again, lacking a precise internal model, the brain will have difficulty moving the arm accurately and even in maintaining the arm in one desired location. There may be an overall drop in muscle output and an inability to stiffen or maintain a position, but there can also be overshoots in which too much muscle force is applied. These difficulties in control arise because of a faulty internal model. Damage to regions thought to be essential for computing the body schema, such as area 5 in the monkey posterior parietal cortex or the superior parietal lobule in humans, can result in similar motor control deficits (Wolpert et al., 1998).

One aspect of an internal model that is sometimes misunderstood is the extent to which it is descriptive of the current condition, and not just prescriptive about how to accomplish a goal. With a description of the current state of the body, it is possible to plan many possible subsequent movements in a flexible manner depending on task goals. One could even argue that all internal models in the brain, including sensory models, are control models. When the visual system constructs a representation of a coffee cup on the table, that internal model serves as a useful description from which a range of possible actions can be planned – whether to reach for the cup, to avoid hitting the cup while reaching past it, to aim a penny into the cup in a game of toss, or whatever other action is task appropriate. A visual model is a model of the outside world such that the brain can exercise better control over the outside world. The body schema is a model of the body such that the brain can exercise better control over the body.

We argue that this relationship – between a real thing, the brain’s representation of that thing, and the successful control of that thing – can be fruitfully applied to understanding the relationship between attention and awareness. In this perspective, awareness is an internal model of attention useful for the control of attention.

One important caveat is helpful to keep in mind. A model of attention might have more than one purpose. Attention, after all, is one of the main drivers of behavior. What you attend to, you are more likely to react to. What you do not attend to, you are very unlikely to react to. Therefore a model of attention could help in predicting one’s own behavior. For example, if you have any intuitive understanding of attention, of its dynamics and consequences for behavior, and if you are concerned about your diet, then you know not to stand all night next to the dessert tray at a party. Out of sight, out of mind – this maxim is essentially about the dynamics of attention. A model of attention could also be of great use in predicting the behavior of other people or animals. If you can reconstruct someone else’s attentional state – what that person is attending to and what the consequences of attention typically are – then you can gain predictive power. If you are on safari and a lion is paying close attention to you, get back in the car. These are general, potential uses of an attention schema. The following sections focus on one specific function – using an attention schema to help in the efficient control of attention.

We argue that because attention is such a complex and variable process, because a brain must control its own attention, and because an internal model is essential for efficient control, the brain is almost certain to have an attention schema – an internal model of attention. We also argue that an internal model of attention, if it is to be a useful model, ought to have the properties we normally ascribe to subjective awareness. *A creature with an attention schema should be a creature that concludes it is aware.*

We view the control problem of directing and regulating attention through top–down biasing signals as having much in common with the control of the body. Attention, much like the body, can be perturbed by external forces. The arm can be moved by forces outside the body. Just so, the state of the brain’s signal competition can be influenced by especially intense or salient stimuli. These external influences need to be accounted for in attempting to control attention. Unexpected influences on the state of the brain’s internal signal competition need to be registered in order to provide the proper set of biasing signals to control and, if necessary, enhance or suppress these influences. We therefore suggest that a simplified model of the process and current state of attention, an attention schema, would be a useful feature of a system concerned with controlling attention.

What exactly would an attention schema depict, and what would it not depict? It may be helpful here to compare it again to the body schema. The body schema does not depict the mechanistic details that underlie the structure and dynamics of the body. It does not depict specific bone structure, muscle insertion points, or the molecular basis of muscle contraction. The brain has no need for that kind of detail in order to be able to control movement of the body. Just so, the hypothesized attention schema would not depict the mechanistic details that make attention possible within the brain. The attention schema would not depict synapses, neurons, lateral inhibition, or electrochemical signals. The brain has no need to model its own processes in that kind of physical detail. Instead, the attention schema would depict something physically incoherent, a process without a physical manifestation – a mental possession or experience of something that empowers one to react to the item. The model would depict a phenomenon that cannot possibly be understood in terms of physical mechanism because it lacks any information about or acknowledgment of physical mechanism.

Why would the brain compute such an incomplete model of its own processes? Because that is all that is needed for the model to be useful. Just as the body schema does not need to represent the mechanistic and cellular details of the body in order to keep track of its general structure and current configuration, a detailed, complete, neuroscientific account of attention is not necessary for keeping track of the current state and general dynamics of attention. The attention schema theory is therefore capable of providing a potential answer to the question of why we are so confident in the existence of an apparently non-physical property, subjective awareness, why we so reflexively attribute it to ourselves – and why scientists are traditionally so stumped to explain a phenomenon that is, almost by definition, without a physical basis. According to the theory, these judgments reflect the information contained in a model that represents the basic features of the process of attention but without any of the details that would be necessary to understand it in terms of physical mechanism. The theory also provides an answer to the question of whether or not subjective awareness serves a useful purpose or is merely an epiphenomenon. It suggests that the model described above would be of great utility, at the very least, in the top–down control of attention.

## Evidence Supporting the Attention Schema Theory: 1. Attention and Awareness are Highly Correlated but Dissociable

**FIGURE 3:**
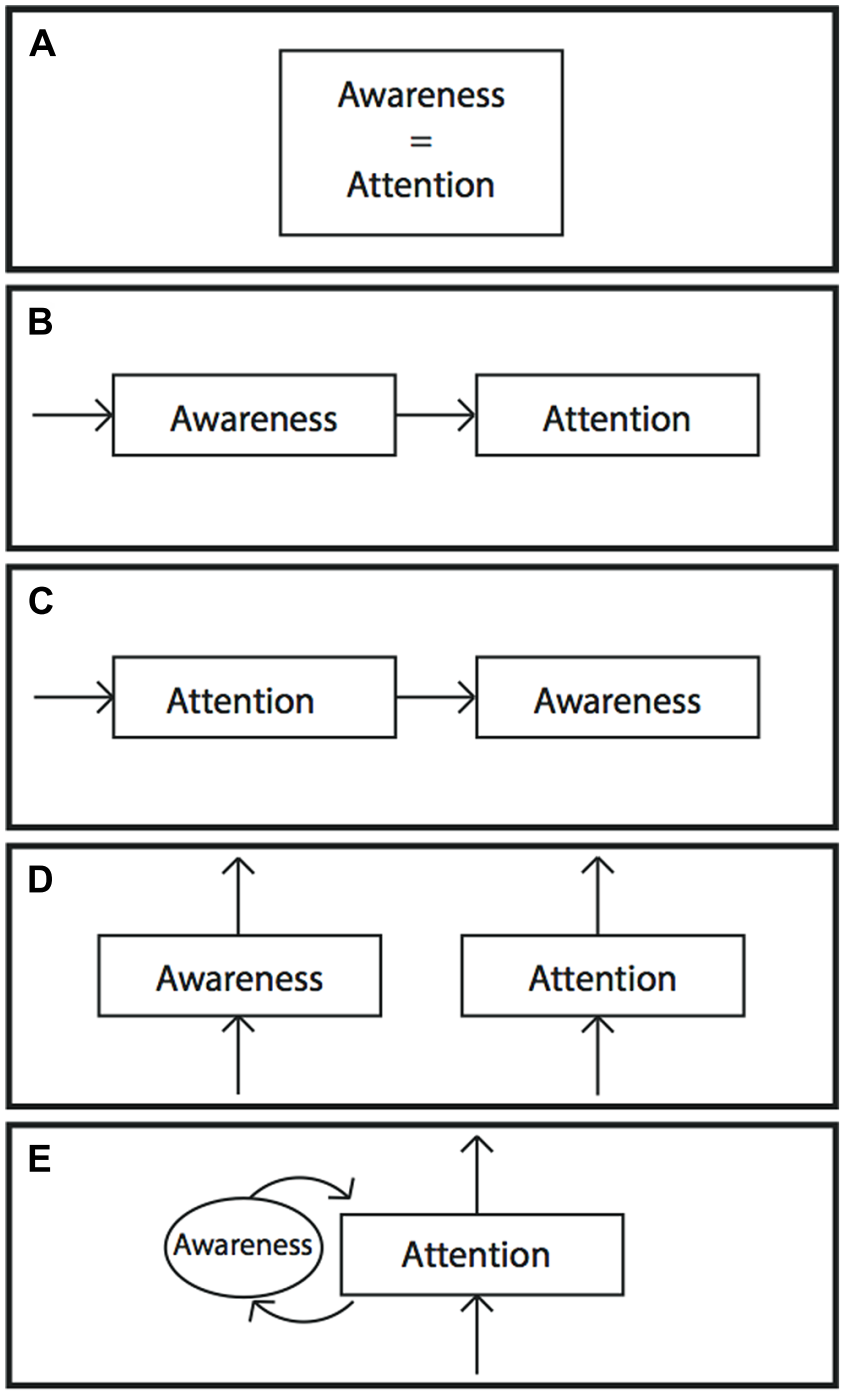
**Five possible hypotheses about the relationship between awareness and attention. (A)** Awareness and attention are the same. **(B)** Awareness precedes attention. One must become aware of something before attending to it. **(C)** Attention precedes awareness. One must attend to something before it can enter awareness. **(D)** Awareness and attention are independent processes in the brain. **(E)** The hypothesis proposed in the present article. In the attention schema theory, awareness is part of the control machinery for attention. It is the internal model of attention, the attention schema. Without awareness, attention is still possible but suffers from deficits in control.

The attention schema theory posits a specific relationship between attention and awareness. To assess the plausibility of the theory, therefore, it is useful to review the extensive previous literature on the relationship between attention and awareness. Clearly, attention and awareness are related. **Figure 3** shows some of the many hypothesized relationships between them.

One example of the close link between awareness and attention is inattentional blindness. Directing top–down attention to one particular stimulus can prevent a person from becoming subjectively aware of other stimuli (Mack and Rock, 1998; Simons and Chabris, 1999; Most et al., 2001). Paying attention to a basketball as it is passed from player to player in a video renders most people totally unaware of the bizarre and salient stimulus of a person in a gorilla suit walking across the basketball court (Simons and Chabris, 1999). This relationship between attention and awareness is so tight, many have argued that there is no difference between them and that ‘attention’ and ‘awareness’ may ultimately refer to the same neuroscientific phenomenon (Posner, 1994; Merikle and Joordens, 1997; Mole, 2008; De Brigard and Prinz, 2010).

However, many studies now suggest that it is possible to dissociate attention from awareness. Some of the earliest demonstrations of attention without awareness came from the study of a patient who suffered from blindsight. Experiments on this patient indicated that spatial attention could be directed to specific locations in the ‘blind’ field of space. This spatial attention improved the patient’s performance on visual tasks at those locations, despite a total lack of subjective awareness of the visual stimuli (Kentridge et al., 1999, 2004).

Subsequent studies showed a similar dissociation between awareness and attention in normal subjects. When a person is not subjectively aware of a stimulus because the stimulus is briefly presented or masked, the stimulus can still draw attention in an automatic, bottom–up manner (McCormick, 1997; Lambert et al., 1999; Ivanoff and Klein, 2003; Lamme, 2003; Woodman and Luck, 2003; Ansorge and Heumann, 2006; Jiang et al., 2006; Kentridge et al., 2008; Hsieh et al., 2011; Norman et al., 2013). Some studies even suggest the possibility of attention in the absence of objective awareness, a more stringent category than subjective awareness, though there may be some controversy about this result (Jiang et al., 2006; Kentridge et al., 2008). Many of these examples are in the domain of spatial attention (McCormick, 1997; Kentridge et al., 1999; Lambert et al., 1999; Ivanoff and Klein, 2003; Woodman and Luck, 2003; Kentridge et al., 2004; Ansorge and Heumann, 2006; Jiang et al., 2006; Hsieh et al., 2011), but examples also exist in other feature dimensions (Kentridge et al., 2008; Norman et al., 2013).

This set of findings presents a confusing picture. Attention and awareness are closely linked, and yet are dissociable. What exactly is the relationship between them? The attention schema theory provides a simple, logical explanation for this relationship. The proposed relationship is diagrammed in **Figure 3E**. In this theory, awareness is part of the control mechanisms of attention. Attention and awareness are not the same thing, but they do normally covary. Their close correspondence is the result of a well-functioning model. Awareness successfully tracks attention because it is an internal model of attention. But the brain’s internal models are never perfect. Indeed, errors in internal models may be relatively common. Almost the entire literature on the body schema is dedicated to the study of illusions, or errors, in the body schema. These internal models are not evolved to be perfect, but to be quickly computable on minimal information and to be useful most of the time. Awareness then, if it is a model of attention, would be expected at least occasionally to become dissociated from attention. These dissociations are generally reported in tasks that involve very dim or masked stimuli at the threshold of detection. It is in that gray area that the internal model of attention seems to fail. An analogous case would be when proprioceptive signals from the body are very weak or masked, the body schema has difficulty updating based on those signals and the body schema loses a clear representation of the position of limbs.

While it is now clear that attention is possible without awareness, a more controversial question is whether awareness is possible without attention. Some recent studies have suggested that this dissociation might be possible (Li et al., 2002; Reddy et al., 2004, 2006; see Koch and Tsuchiya, 2007 for review), though others have disputed the claim (Cohen et al., 2012). Though the issue is unresolved, it is worth asking whether that condition is consistent with the attention schema theory. The answer is not very clear. The theory may allow for awareness without attention, but there are several caveats.

Suppose that a stimulus activates perceptual machinery in the brain, such as the case of the apple in **Figure 1A**. However, suppose in this case the stimulus is not attended – other representations dominate processing in these brain regions. It may still be possible for the internal model of attention to be erroneously linked to that stimulus representation, incorrectly indicating that attention has been drawn by the stimulus. In that case, the “S+A+V” in **Figure 1B** would be the result of an error in the system that incorrectly attached the model of attention to a stimulus representation that is not, actually, commanding any attention. According to the attention schema theory, this is all that would be necessary to have awareness without attention.

On the other hand, two issues work against this possibility. First, it has been proposed that attention is necessary to bind together the different components of a representation (Treisman and Gelade, 1980). If that is the case, then without attention to stimulus X, it may be impossible for the representation of X to be bound to other information, such as the information in the attention schema. In that case, the construct of awareness would never be integrated with the representation of X, and the brain would not contain sufficient information to conclude or report that it was aware of X.

Second, without attention, a stimulus representation has lower signal strength and is much less likely to impact behavior. Therefore, it is much less likely that a person would be able to actively report on it. Thus, in the attention schema theory, even if the brain ever did build the construct of awareness and erroneously attach it to an unattended stimulus, it is unlikely that someone would be able to explicitly report the condition. Subjective report would be hampered. In the attention schema theory, while attention without awareness is predicted, awareness without attention is more complex and seems less likely, but is not entirely ruled out.

## Evidence Supporting the Attention Schema Theory: 2. Attention is Less Well-Controlled in the Absence of Awareness

An increasingly common view is that attention and awareness have no principled relationship with one another. They are orthogonal functions. This view is based on the reports of dissociation between attention and awareness described above. The attention schema theory, in contrast, posits a specific, principled relationship between attention and awareness. In this proposal, attention is a complex and necessary process in the brain, and awareness is a simplified model of that process. That model may serve many functions, but one function is to assist in the control of attention. This proposed relationship leads to a unique empirical prediction. Without awareness, attention should still be possible but should be less effectively controlled.

In the same way, if the brain lacks a clear internal model of the arm, such as in the case of anesthesia of the arm, then the control of the arm is still possible but is less effective. Without knowing where the arm is or how it has been perturbed by external forces, the system cannot easily accomplish goal-directed movement. Just so, in the attention schema theory, if the brain is allocating attention to item X but unaware of X, it has made an error in its internal model of attention, failing to register that its attention has been perturbed by X. In that case, the top–down control of attention should be compromised. The scenario is probably easiest to imagine in the domain of spatial attention, especially because of the direct analogy that one can draw with the model-based control of the body in space, but the same prediction could apply, in principle, to any domain in which attention operates. Attention could be subliminally drawn to a particular color while a person is trying to make a discrimination concerning stimuli of a different color, or attention could be subliminally drawn to a particular stimulus feature to the detriment of successfully processing another feature of that stimulus. Regardless of the informational domain, the general prediction is the same: without awareness, attention should still be possible but should suffer a deficit in control.

This consequence of the attention schema theory is consistent with at least some previous evidence. In one study (McCormick, 1997), participants performed a spatial attention experiment closely resembling the classic attention paradigm developed by Posner (1980). Subjects fixated at a central location on a screen and a cue was presented briefly to one side or the other. If the cue was on the left, it indicated to the subject to attend to the right and respond to a subsequent target stimulus. If the cue was on the right, it indicated to the subject to attend to the left and respond to the subsequent target stimulus. This anti-correlated cue provided a useful test. Naturally the cue drew automatic, bottom–up attention to itself. McCormick found that, when participants were aware of the cue, they were capable of overcoming this initial, pre-potent attentional effect. They were able to pull attention away from the cue and direct it to the opposite side where the target was most likely to appear. In contrast, when participants were unaware of the cue, the bottom–up, pre-potent effect dominated, biasing attention to the side that the cue appeared, ultimately to the detriment of task performance on these trials. This experiment demonstrates that, with awareness of the cue, attention to that cue could be controlled in a top–down manner. Without awareness of the cue, attention to the cue could no longer be controlled in a top–down manner. Attention was, in effect, stuck on the location of the subliminal stimulus, and the subject no longer had the ability to control attention to the extent of prying it away from that spot.

Another striking example was reported by Tsushima et al. (2006). Subjects performed a centrally presented letter discrimination task while a distracting dot motion stimulus was presented in the periphery. Performance on the letter task was actually better when the subjects were aware of the distracting motion, and performance was most impaired when the distracting motion was subthreshold and subjects were unaware of it. In other words, when participants were aware of the distracting motion, they were capable of controlling their attention, keeping it from the distracting stimulus and on the task. But when subjects were unaware of the distracting motion, they were no longer able to suppress their attention to it, leading to a disruption in the central task.

The effects from these two studies are highly counterintuitive. In both instances, a subliminal stimulus had a greater effect on attention than a consciously perceived stimulus. How is it possible for an unconscious stimulus to have a greater effect than a conscious one? We believe that the attention schema theory provides a simple explanation for this counterintuitive phenomenon. In the theory, awareness is part of the control mechanism for attention. Without awareness, attention is still possible, but the brain in essence lacks knowledge about its state of attention and therefore cannot properly regulate that attention. If attention is directed at stimulus X in the absence of awareness of stimulus X, the brain has no internal knowledge that it is attending to X and therefore the control mechanism cannot easily withdraw that attention from X, or take that attention on X into account when adjusting attention to a different stimulus Y. As a result, the top–down control of attention to X, to Y, or to other stimuli is not as efficient. In that situation, stimulus X has a less well-controlled effect on behavior than it would otherwise.

An interesting corollary to these findings comes from the social psychological literature. Apparently if you are unaware of a stimulus, it can sometimes have a greater undesirable effect on your social judgments, whereas if you are aware of the stimulus, you can to some extent mitigate its social biasing effect. For instance, Bargh et al. (1996) showed that presenting subliminal images of black faces to white participants can trigger an automatic negative association or affect. This appears to be the case even for participants who are explicitly opposed to racism and affirm the importance of egalitarian values (Dovidio et al., 1997). Similar effects have been reported in other experiments (Devine, 1989; Lowry et al., 2001; Eberhart et al., 2004).

One such experiment (Devine, 1989) attempted to compare the effects of consciously versus unconsciously perceived manipulations. When participants were subliminally primed with words that activated stereotypes of black people, they showed subsequent stereotyping biases regardless of whether the participants scored highly on explicit measures of racism. Explicit opposition to racism did not preclude the activation of racial biases when these biases were subliminally activated. However, when participants were given a task in which stereotypes were consciously manipulated, those who scored low on explicit measures of racism were able to inhibit the undesirable, biased responses. When aware of a stimulus, participants could regulate their behavior in line with their explicit views about race. When unaware of the stimulus, that top–down control disappeared, such that even participants who were not explicitly prejudiced could still be induced to behave in a biased manner.

Taken together, the studies summarized above are highly suggestive of the specific relationship that we propose regarding attention and awareness. In the absence of awareness of a stimulus, the effects of that stimulus on attention and therefore on behavior cannot be regulated in line with goals or task demands as well as when the stimulus is consciously perceived.

On the surface, there is a seeming triviality to this pattern of results. It aligns with the most common intuitions about consciousness. If you’re not conscious of something, we all know from experience that you can still sometimes find yourself reacting to it. Things go on under the surface of consciousness. And in that condition, when you react unconsciously, you have no control over that reaction. It just pops out. How can you control it, if you’re not conscious of it? Everyone knows this to be true. It is intuitively obvious.

We would argue, however, that this intuitive understanding is entirely non-explanatory. It is folk psychology in which, with some circularity, consciousness is the thing in me that, when conscious of something, allows me to consciously choose how to react to that thing. The present theory provides a specific, underlying explanation for these common folk intuitions. At the root of these effects is awareness as a model of attention. Without awareness, without that model of attention, the control of attention and therefore of behavioral reaction is poor.

## External versus Internal Awareness

A key challenge for theories of the brain basis of awareness is to explain how we can become subjectively aware of both internal and external content. Internal content, such as abstract thoughts and emotions, and external sensory content seem like radically different entities, and yet both can inhabit our subjective awareness. How is this possible?

The attention schema theory is well-equipped to answer this question. The attention schema theory holds that subjective awareness is the brain’s simplified model of its own process of attention. But attention is domain general. The most commonly studied variety of attention is spatial attention, but much work has also been done on attention in other stimulus dimensions. Attention has been shown to operate in the domain of color (Anllo-Vento et al., 1998), motion (O’Craven et al., 1997), time (Coull and Nobre, 1998), form (Wojciulik et al., 1998), and a large list of increasingly exotic stimulus dimensions. Much work has also been done on attention as it operates between competing stimulus dimensions (Maunsell and Treue, 2006). Indeed, it is tempting to conclude that attention is a universal feature of brain function, acting within and between any dimensions in which the brain can process information.

A growing body of work has focused on attention toward internal versus external content. The general conclusion is that attention can be focused on internal content as well as external content, and that many of the same dynamics apply (Chun et al., 2011). Especially salient emotions, memories, and even abstract thoughts can sometimes suddenly dominate brain processes in much the same way that a salient visual or auditory stimulus might. Attention can also be biased in a top–down manner toward specific internal content. The representation of particular memories, task rules, emotional states, or goals can be biased so that they are more deeply processed and therefore more likely to determine and guide behavior.

According to the attention schema theory, the brain constructs a simplified model of the complex process of attention. If the theory is correct, then the attention schema, the construct of awareness, is relevant to any type of information to which the brain can pay attention. The relevant domain covers all vision, audition, touch, indeed any sense, as well as internal thoughts, emotions, and ideas. The brain can allocate attention to all of these types of information. Therefore awareness, the internal representation of attention, should apply to the same range of information.

## Conclusion

We argue that the attention schema theory provides a possible answer to the puzzle of subjective experience. The core claim of the theory is that the brain computes a simplified model of the process and current state of attention, and that the content of this model is the basis of subjective reports. According to the theory, subjective reports such as ‘I am aware of X’ involve the following steps. Stimulus X is encoded as a representation in the brain, competing with other stimulus representations for the brain’s limited processing resources. If stimulus X wins this signal competition, resulting in its being deeply processed by the brain, then stimulus X is attended. According to the theory, an additional step is needed to produce a report of subjective awareness of stimulus X. The brain has to compute a model of the process of attention itself. Attention is, in a sense, a relevant attribute of the stimulus. It’s red, it’s round, it’s at this location, and it’s being attended by me. The complex phenomenon of a stimulus being selectively processed by the brain, attention, is represented in a simplified model, an attention schema. This model leaves out many of the mechanistic details of the actual phenomenon of attention, and instead depicts a mysterious, physically impossible property – awareness. The brain reports the presence of awareness of the stimulus because it is reporting the contents of its internal models. The brain can report only the information available to it through its internal models.

The theory is partially based on the logic of model-based control. Just as the brain computes a model of the body, the body schema, and uses this model in the control of the body, we suggest that a simplified model of attention, an attention schema, would be useful in controlling attention. Certain predictions about the relationship between attention and awareness follow straightforwardly from this control-theory approach, and at least some of these predictions correspond closely with the existing literature. It is our hope that future experiments will provide further tests, and in the process provide an answer to the many questions surrounding subjective experience and the purposes it might serve in the functioning of the brain.
